# Bacterial filamentation during urinary tract infections

**DOI:** 10.1371/journal.ppat.1010950

**Published:** 2022-12-01

**Authors:** Charlotte Abell-King, Ariana Costas, Iain G. Duggin, Bill Söderström

**Affiliations:** Australian Institute for Microbiology and Infection, University of Technology Sydney, ULTIMO, Australia; Duke University School of Medicine, UNITED STATES

Urinary tract infections (UTIs) are established when a uropathogenic microbe enters the urinary tract, avoids the immune system, and initiates colonization and infection that damages the host [1–3]. They are among the most common bacterial infections with many resulting in antimicrobial resistance (AMR)-related deaths [4]. A study over 10 years, following 700,000 community-acquired UTIs, found that uropathogenic *Escherichia coli* (UPEC) was the causative agent in 70% of cases, with *Klebsiella pneumoniae* and *Proteus mirabilis* in 10% and 5% of cases, respectively [5]. Estimations have suggested that at least 150 million people experience a UTI annually [6]. Certain groups are disproportionately at risk, with the majority (approximately 60%) of women experiencing at least one UTI in their lifetime [7–9]. Recurrent UTIs (rUTIs) are also prevalent: With up to 25% of patients experience another infection in months after apparently successful antimicrobial treatment, partly due to the rise of antibiotic-resistant UTI pathogens [5,10]. It is not yet clear how rUTIs are so persistent, but key to understanding this may be in the specific bacterial lifestyles and infection cycles, where bacterial filamentation and L-form formation have been suggested to play important roles [11,12]. While L-form formation may be an important reservoir for persistent UTIs, it is outside the scope of this text, as in this Pearls, we focus on what is known about bacterial filamentation and reversal in a bladder environment.

## The UPEC morphology cycle

**Fig 1:**
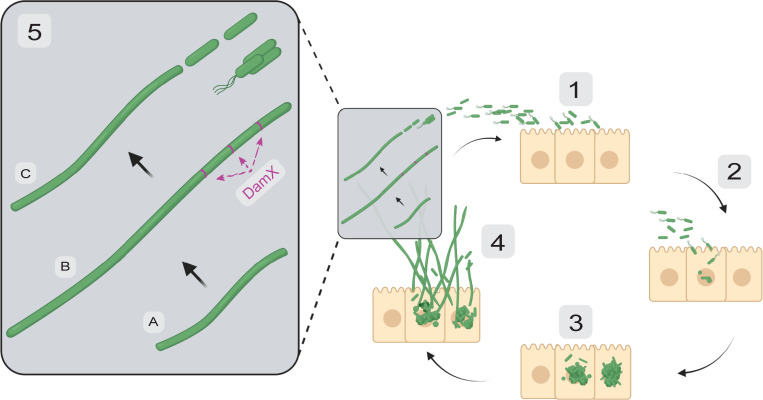
Simplified schematic of the UPEC morphology cycle during UTIs. **(1**) Rod-shaped bacteria adhere to BECs. (**2**) Invasion via endocytosis. (**3**) Rods undergo shape changes to a cocci-like form and densely cluster together in biofilm-like IBCs. In a second step, a subpopulation of the bacteria reinitiate growth, without dividing, to become filamentous. The exact molecular ques regulating this are unknown, but the cell division protein DamX is essential for filamentation [38]. (**4**) The growth of IBCs and filaments overwhelms the bladder cell, which ruptures whereby UPEC of various morphologies are expelled. (**5**) Exfoliated filaments (**A**) can continue to elongate to 100s of micrometers (**B**) before the cell division machinery is “switched on” and reversal is initiated (**C**). DamX also has a function during reversal (filament division); DamX tagged with a fluorescent protein forms stable rings at division sites along the filaments [31,38]. Daughter cells are pinched off from the mother filament at an increasing rate during the early stages of reversal, which would allow reinitiation of the infection cycle by invasion of noninfected bladder cells (**1**). BEC, bladder epithelial cell; IBC, intracellular bacterial community; UPEC, uropathogenic *Escherichia coli*; UTI, urinary tract infection.

UPEC displays a distinct pathogenesis cycle in a bladder environment (Fig 1) [13,14]. These rod-shaped bacteria use cell surface fibers to adhere to superficial umbrella bladder epithelial cells (BECs), before invading the cytoplasm [15], where they develop as biofilm-like intracellular bacterial communities (IBCs) comprising many bacteria that appear as coccoid shapes organized in condensed bacterial clusters [16,17]. Further development of IBCs can result in their occupancy of most of the infected cell, eventually resulting in its rupture and dispersal of the bacteria. The dispersal stage involves at least two types of bacterial differentiation, where a subset of cells become rod shaped and motile, and others will stop dividing and grow into highly elongated bacterial filaments. The full picture of molecular cues regulating bacterial differentiation in UTI is currently unknown. Here we will consider the UPEC filamentation process, referred to as infection-related filamentation (IRF), as a remarkable example of bacterial differentiation and its possible functions in UTI. The regulation of IRF has partly been attributed to the cell division regulating gene *sulA*. *sulA* has been suggested to play a role in filamentation as early result indicated that a UTI89Δ*sulA* strain was unable to filament in a murine model [2]. Another possible contributor to the regulation of filamentation is innate immune system itself as in mice lacking the TRL4 receptor is filamentation not observed [2,18,19].

It is understood, however, that IRF is likely to take place due to environmental pressures experienced in the bladder, e.g., innate immune effectors and weakly acidic urine [18,20,21]. It is currently not clear how urine is regulating the elongation response as filamentation is initiated inside the epithelial cells, but it has been observed that both urine composition and acidity is essential for UPEC filamentation in in vitro infection models [21]. Long filaments, up to several hundreds of micrometers in length, have been observed both in vivo and in vitro [2,20]. While UPEC has the most studied infection cycle, filamentation has also been observed for other UTI-associated pathogens, such as *K*. *pneumoniae* and *Pseudomonas aeruginosa* during bladder infections in murine models [22–28]. Although the morphology cycle during infection has been described, some key questions remain, including how do morphology transitions occur and what is their purpose?

## Is filamentation a bacterial dispersal strategy during UTIs?

Bacterial filamentation occurs when advanced IBCs or biofilms of UPEC are exposed to weakly acidic urine [20,21,29]. Interestingly, a synthetic urine of the same pH failed to trigger filamentation in the cell culture model of infection, and the exact urine components and UPEC response pathways responsible are still unknown. Additional host factors might also play direct or indirect roles, as filamentation was not detected in mice lacking the TLR-4 receptor that triggers an immune response to gram-negative bacteria [2]. Filamentation is believed to function at least in part as an innate defense mechanism against the human immune system [2]. It is believed that both their size and shape inhibit uptake by macrophages; however, this is not yet resolved as early experiments using plastic particles showed that shape rather than size was a determinant for uptake, as “non-rod”-shaped objects were less likely to be phagocytosed [30]. There have also been multiple reports that filaments from in vivo models are resistant against internalization and thus killing by phagocytes [2,18,28].

But could filamentation also be a means for efficient dispersal? Filaments still form in UPEC biofilms in response to urine even in the absence of host cells [29]. Furthermore, filaments have been seen to extend 100s of micrometers from human BECs during in vitro model infections [21]. After dispersal, filaments have been observed to elongate at a high rate, 1.8 μm min^−1^, translating to half a “rod-length” per minute (assuming average rods are 3.5 μm), with an average of 0.55 μm min^−1^, enabling growth of more than 100 μm before initiating reversal back to rods [31]. As a result of the accumulated extra body mass, filaments may have an increased adhesion capacity to host cells and an improved ability to resist the liquid shear forces in the bladder [2,20]. These observations suggest that filamentation could be a deliberate action by UPEC to disperse from the IBC and extend out to reach neighboring cells to maximize infection propagation.

On dispersal, filaments will experience a rapid change of environment, which appears to trigger a coordinated reversal (division) to form rods. As daughter cells pinch off from the mother filaments, they divide at faster rates than typically observed for normal *E*. *coli* binary fission in standard laboratory growth conditions (e.g., 37°C in LB) [31,32]. The current understanding is that filaments cannot invade further BECs but must first revert into many rods, which can individually restart the infection cycle by infecting thus far uninfected epithelial cells, making reversal a crucial part of the bacterial morphology cycle during UTIs [33].

## Reversal of filaments back to rods: Not regulated as binary fission during vegetative growth?

Binary fission in *E*. *coli* is an extensively studied process mediated by a multiprotein complex organized by the essential division protein, FtsZ, a homolog of the eukaryotic cytoskeletal protein tubulin [34]. During vegetative growth, FtsZ is the first protein to arrive at the division site forming a “proto-ring” around the midcell, with helper proteins including FtsA and ZipA that help anchor it to the inner membrane [35]. There are 12 essential divisome protein recruited to the midcell, forming a structure known as “the divisome” [36]. Divisome maturation is finalized with the arrival of FtsN, which initiates constriction of the cell envelope [37]. The complexity of the essential functions of the divisome provides many potential avenues for regulation.

The regulation of division during IRF is less well understood. It is believed that filament reversal only occurs outside the epithelial cells, as no observation has been reported to support that this process takes place intracellularly. In in vitro infection systems, multiple divisions occur at high temporal rates during reversal (with generation times down towards 10 minutes; [31]), as such one wonders about the state of the cell division machinery during filamentation and the onset of reversal. It is tempting to imagine that parts, if not the whole machinery, could be assembled at multiple locations along the filament body ready to get into action.

Unlike binary fission, where the maturation and stabilization of FtsZ polymers into a Z-ring regulates cell division, FtsZ in filaments forms transient Z-rings, assembling and disassembling multiple times at multiple locations [31]. The formation and dynamics of these transient Z-rings suggest another level of regulation specific to filamentation and reversal. This regulation is currently believed to be provided by DamX through an unknown mechanism [31,38]. During its function in inhibiting division during IRF, DamX remains dispersed throughout the inner membrane; then, during reversal, it condenses into stable rings that always result in a division event [31]. DamX belongs to a group of cell division proteins that are targeted to septal peptidoglycan by a highly conserved sporulation-related repeat domain, or SPOR domain [39]. Interestingly, while deletion of *damX* in nonpathogenic model laboratory *E*. *coli* strains (e.g., K-12) shows no apparent phenotypes, a deletion of the same gene in the model UPEC strain, UTI89, shows an elongation phenotype reflecting a defect in cell division [31], highlighting the importance of the use of pathogenic model strains.

## Concluding remarks

Bacterial cell division has been intensely studied for over 20 years, but recent advances in infection models show how relatively little we know about this process in a disease setting. The dynamic molecular shifts occurring in filamentation and their divergence from the characteristic cell division seen in vegetative growth demonstrate an essential role of SPOR domain proteins in regulating filamentation and its reversal. While filament division appears to be performed by the same machinery as in binary fission, the regulation appears to differ in key aspects and warrants further study. Apart from UPEC, other bacteria also undergo filamentation in various infectious and environmental settings, but the molecular regulation for this behavior is not well understood [24–27]. Our hope is that in vitro cell culture models or microfluidic “lab-on-a-chip” models will allow new insights into the regulation of cell division and morphology of pathogenic model strains in UTIs and other prevalent AMR-related infections, generating new knowledge that will inform the development future therapies.
